# A new defense in the battle of the sexes

**DOI:** 10.7554/eLife.50140

**Published:** 2019-08-16

**Authors:** George L Sutphin

**Affiliations:** 1 Department of Molecular and Cellular BiologyUniversity of ArizonaTucsonUnited States; 2 BIO5 InstituteUniversity of ArizonaTucsonUnited States

**Keywords:** mating, aging, insulin signaling, mTOR, sperm-sensing, *C. briggsae*, *C. elegans*

## Abstract

Young *Caenorhabditis elegans* hermaphrodites use their own sperm to protect against the negative consequences of mating.

**Related research article** Shi C, Booth LN, Murphy CT. 2019. Insulin-like peptides and the mTOR-TFEB pathway protect *C. elegans* hermaphrodites from mating-induced death. *eLife* **8**:e46413. doi: 10.7554/eLife.46413

**Related research article** Booth LN, Maures TJ, Yeo RW, Tantilert C, Brunet A. 2019. Self-sperm induce resistance to the detrimental effects of sexual encounters with males in hermaphroditic nematodes. *eLife* **8**:e46418. doi: 10.7554/eLife.46418

In popular culture, the phrase 'the battle of the sexes' conjures images of the complexities of dating, discussions about gender or, for some, a movie about the ultimate tennis grudge match. In the animal kingdom, the battle of the sexes can be much more visceral, and often deadly. Consider the roundworm *Caenorhabditis elegans:* hermaphrodite worms can reproduce through self-fertilization or by mating with male worms, but sexual interaction between males and hermaphrodites shortens the lifespan of both (Maures et al., 2014; Van Voorhies, 1992).

Self-fertilizing hermaphrodites enjoy a substantial post-reproductive lifespan, whereas those that mate with males typically only survive until they produce the last of their offspring (Shi and Murphy, 2014). The negative impact of sex on the lifespan of hermaphrodite worms is mediated by a number of molecular mechanisms including pheromones, the transfer of seminal fluid, and germline activation (Shi et al., 2017). Now, in two papers in eLife, researchers at Stanford University and Princeton University report that the 'self-sperm' produced by young hermaphrodites protects them from the dangers associated with mating. Each paper describes different signaling pathways involved in providing this protection.

In one paper Anne Brunet of Stanford University and co-workers – including Lauren Booth as first author with Travis Maures, Robin Yeo, and Cindy Tantilert – report that self-sperm protects *C. elegans* against aging by activating the 'sperm-sensing' pathway (Booth et al., 2019). Young hermaphrodite worms that mate have normal lifespans, while old worms die soon after mating. Booth et al. showed that, in young worms, normal lifespan after mating was the result of activating of the sperm-sensing pathway, which repressed the transcription factor CEH-18 and the ephrin receptor VAB-1 (Figure 1). In old worms, self-sperm was depleted and could no longer repress these proteins, leading to death. Booth et al. also investigated whether other species of roundworm were protected by self-sperm, discovering that *Caenorhabditis briggsae* shared this characteristic. However, this protection evolved relatively recently and independently from that of *C. elegans*, which suggests that roundworms continue to experience pressure from natural selection related to sexual interaction.

**Figure 1.**
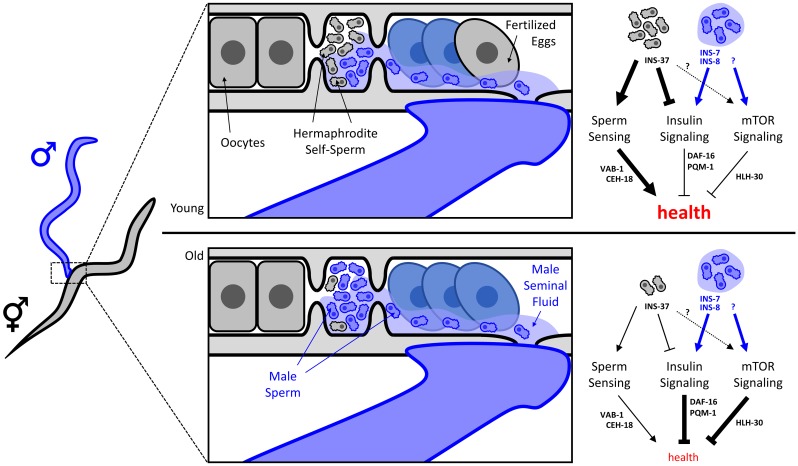
How self-sperm protects young hermaphrodite *C. elegans* from the damaging effects of seminal fluid during mating. When male and hermaphrodite *C. elegans* roundworms mate, male sperm (blue) and seminal fluid (pale blue) mix with hermaphrodite self-sperm (grey). Nearly all oocytes are fertilized by male sperm rather than self-sperm following mating. The seminal fluid and self-sperm act on three signaling pathways that influence the health and longevity of *C. elegans*: an active sperm-sensing pathway is positive for the health of the roundworm, whereas active insulin and mTOR signaling pathways have negative effects on its health. Seminal fluid damages the health of roundworms because it activates both the insulin signaling pathway (through INS-7 and INS-8) and the mTOR signaling pathway (through a yet-to-be-discovered mechanism). Self-sperm, on the other hand, is good for the health of hermaphrodite roundworms because it activates the sperm-sensing pathway, and represses both the insulin signaling pathway (through the insulin peptide INS-37) and the mTOR signaling pathway (again through an unknown mechanism). Young hermaphrodites (top) have more self-sperm than old hermaphrodites (bottom), so they benefit from increased sperm-sensing signaling as well as reduced insulin and mTOR signaling. Old worms have less self-sperm to counter the adverse effects of seminal fluid, so they succumb to the negative consequences of insulin and mTOR signaling and die rapidly. The sperm-sensing pathway improves longevity by repressing the ephrin receptor VAB-1 and the transcription factor CEH-1. Activation of mTOR and insulin signaling removes two transcription factors that promote longevity (DAF-16 and HLH-30) from the cell nucleus, and translocates a transcription factor that reduces longevity (PQM-1) into the nucleus.

In the other paper Coleen Murphy of Princeton University and colleagues – including Cheng Shi as first author and Lauren Booth – explore the influence of male seminal fluid on the insulin and the mTOR signaling pathways (Shi et al., 2019). In particular, they find that the insulin signaling pathway is activated by two peptides present in male seminal fluid (INS-7 and INS-8) and repressed by INS-37, a peptide found in self-sperm. As aging hermaphrodite worms deplete their supply of self-sperm, they lose the protective repression of insulin signaling and only experience the mating-induced activation. Shi et al. also find that unknown molecules in male seminal fluid activate the mTOR signaling pathway, driving the removal of the pro-longevity transcription factor HLH-30 from the nucleus, which leads to mating-related death (Figure 1).

The work at Stanford and Princeton has broader relevance to our understanding of the molecular mechanisms of aging. To take one example, the drug fluorodeoxyuridine (FUdR) is widely used to prevent *C. elegans* reproduction during aging studies because it inhibits the production of eggs and sperm in hermaphrodites. The drug affects lifespan differently depending on the age of the worms (Wang et al., 2019), while also enhancing some pro-longevity treatments (Anderson et al., 2016). Understanding the relationship between FUdR, self-sperm, and signaling pathways involved in aging may lead to the development of new methods to study aging in *C. elegans*.

Sexual interactions also affect health in mammals, albeit in a less dramatic fashion. For example, the presence of male mice is sufficient to increase female body weight and stress while accelerating the onset of puberty (Garratt et al., 2016; Flanagan et al., 2011). Conversely, male mice maintained in the presence of females remain fertile substantially longer than those who live alone (Schmidt et al., 2009). While the specific role of self-sperm is not directly relevant to mammals, this work places evolutionarily conserved longevity pathways squarely at the intersection of sexual interaction and long-living invertebrates. Will the same be true for mammals?
